# Metabolic Engineering of *Pseudomonas putida* KT2440 for β-Nicotinamide Mononucleotide Biosynthesis from Glucose and Aspartate

**DOI:** 10.3390/microorganisms14091877

**Published:** 2026-08-24

**Authors:** Luna Gao, Lin Wei, Siqi Wang, Jun Li, Jingli Liu, Zhao Guo, Zhi-Min Li, Zhimin Li

**Affiliations:** 1 Jiangxi Engineering Laboratory for the Development and Utilization of Agricultural Microbial Resources, College of Bioscience and Bioengineering, Jiangxi Agricultural University, Nanchang 330045, China; 2 College of Food Science and Engineering, Jiangxi Agricultural University, Nanchang 330045, China; 3 College of Chemistry and Materials, Jiangxi Agricultural University, Nanchang 330045, China

**Keywords:** β-nicotinamide mononucleotide, *Pseudomonas putida* KT2440, metabolic engineering, NAD^+^ metabolism, transporter engineering

## Abstract

β-Nicotinamide mononucleotide (NMN) is an important intermediate in nicotinamide adenine dinucleotide (NAD^+^) metabolism and has attracted increasing interest as a bioactive compound and biomanufacturing product. In this study, *Pseudomonas putida* KT2440 was engineered for NMN production using a “block–enhance–transport” strategy. Deletion of *nicB* impaired nicotinic acid degradation and resulted in the accumulation of 66.2 μM nicotinic acid in cell extracts, whereas additional deletion of the putative NMN-consuming genes *pncC* and *ushA* did not lead to detectable NMN accumulation. Coexpression of endogenous *pncB* and engineered Francisella tularensis nadE* enabled low-level NMN formation through a Preiss–Handler pathway-based route. By contrast, overexpression of endogenous *nadA*, *nadB*, and *nadC* strengthened precursor supply through the NAD^+^ de novo biosynthetic pathway and resulted in approximately 0.17 mM NMN in cell extracts. Chromosomal integration of an engineered Salmonella enterica pnuC* transporter cassette was associated with pronounced extracellular NMN accumulation. Additional overexpression of genes involved in downstream NAD^+^ metabolism or phosphoribosyl pyrophosphate supply did not considerably improve production, possibly because of metabolic competition or expression burden. The best-performing strain, LW10, produced 1.28 mM extracellular NMN, corresponding to approximately 0.43 g/L, after 96 h of shake-flask cultivation in basal salt medium containing glucose and L-aspartate. These results suggest the feasibility of NMN biosynthesis in engineered *P. putida* KT2440 and highlight the importance of balancing precursor supply, competing reactions, and product transport. Thus, *P. putida* KT2440 represents an alternative chassis for further pathway balancing and process optimization toward fermentative NMN production.

## 1. Introduction

β-Nicotinamide mononucleotide (NMN) is a pyridine nucleotide intermediate that plays an essential role in the biosynthesis and turnover of nicotinamide adenine dinucleotide (NAD^+^). NAD^+^ functions as an indispensable redox cofactor and a substrate for diverse NAD^+^-consuming enzymes involved in energy metabolism, DNA repair, signal transduction, and stress responses [1,2]. Owing to its role as a direct NAD^+^ precursor, NMN has received growing attention in the fields of nutritional science, health-related products, and biomanufacturing [3,4].

NMN can be produced by chemical synthesis, enzymatic catalysis, or microbial fermentation. Chemical synthesis can achieve industrial-scale production but may require multistep reactions, expensive reagents, and challenging stereoselective purification because only β-NMN is biologically active [5,6]. Enzymatic approaches, particularly those using nicotinamide riboside kinase (NRK), offer high substrate specificity and mild reaction conditions. However, enzyme stability, ATP regeneration, and the cost of substrates such as phosphoribosyl pyrophosphate (PRPP) and nicotinamide riboside (NR) remain important limitations [7,8,9].

Microbial fermentation is considered an attractive alternative because it can use inexpensive carbon sources and integrate cofactor regeneration, precursor supply, and product synthesis within a single cellular system [10,11]. Several metabolic pathways have been designed for NMN synthesis in microorganisms. These include (i) the nicotinamide phosphoribosyltransferase (Nampt)-mediated conversion of nicotinamide and PRPP into NMN [12,13,14]; (ii) NRK-mediated phosphorylation of NR [15]; (iii) conversion of nicotinate mononucleotide (NaMN) into NMN using a modified NMN synthetase [16,17]; and (iv) pathways coupled to endogenous NAD^+^ metabolism [18]. Recent studies have substantially improved NMN production in engineered microorganisms, including titers in the gram-per-liter range under optimized fed-batch fermentation conditions [14,19,20,21]. Nevertheless, the development of alternative microbial hosts remains important to expand the available metabolic capabilities and process robustness of NMN-producing cell factories.

*Pseudomonas putida* KT2440 is a well-characterized Gram-negative bacterium widely used in metabolic engineering and synthetic biology. The strain exhibits broad substrate utilization, high tolerance to environmental and chemical stresses, and a versatile central carbon metabolism [22,23]. In addition, a growing toolbox of expression vectors, genome-editing methods, and regulatory elements has facilitated engineering of *P. putida* KT2440 for the production of organic acids, aromatic compounds, polymers, and other value-added chemicals [24].

**Figure 1:**
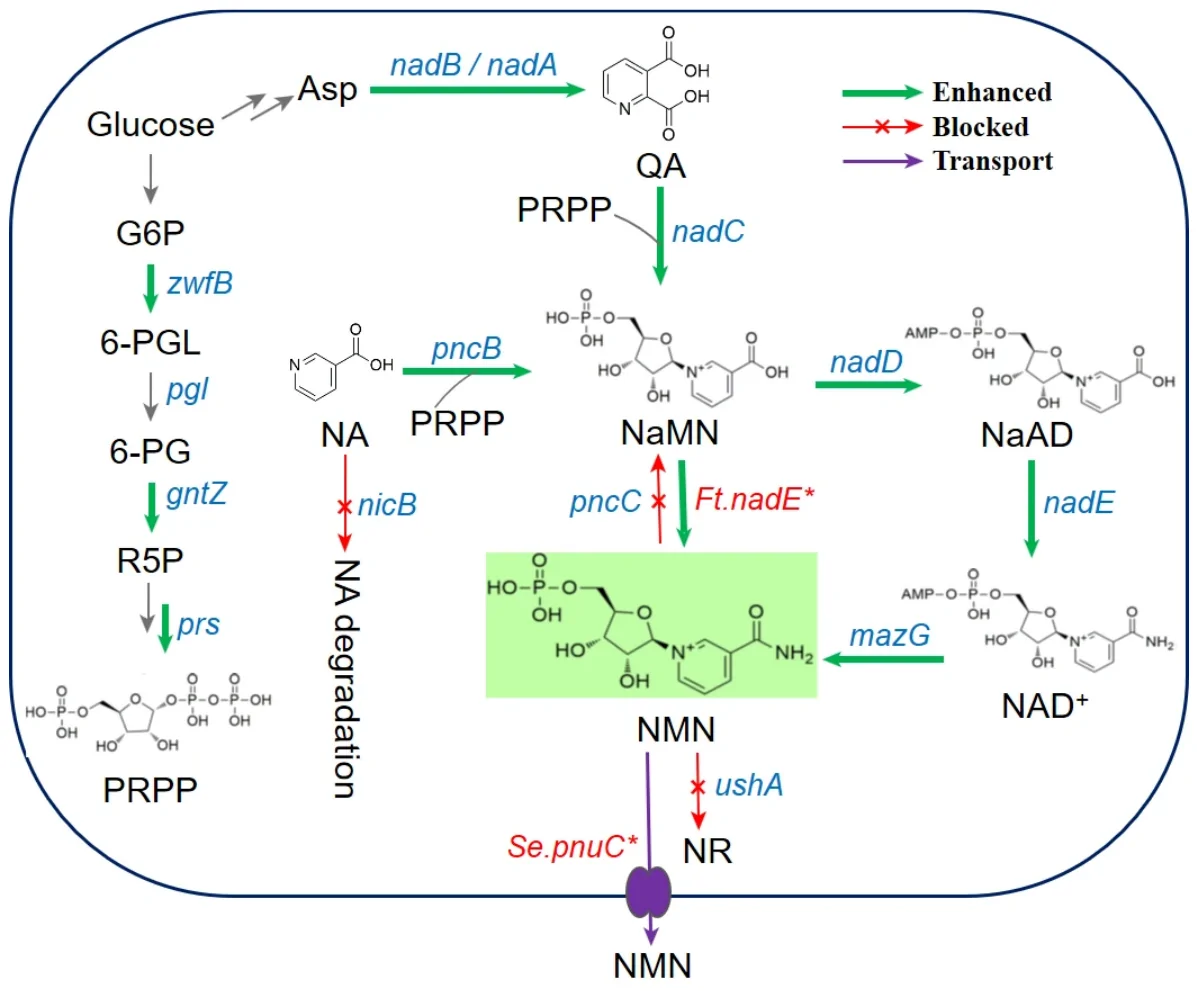
Schematic illustration of NMN biosynthetic pathways in *Pseudomonas putida* KT2440. G6P, glucose-6-phosphate; 6-PGL, 6-phosphogluconolactone; 6-PG, 6-phosphogluconate; R5P, ribose-5-phosphate; PRPP, 5-phosphoribosyl-1-pyrophosphate; Asp, aspartate; QA, quinolinic acid; NA, nicotinic acid; NaMN, nicotinic acid mononucleotide; NaAD, nicotinic acid adenine dinucleotide; NAD^+^, nicotinamide adenine dinucleotide; NMN, nicotinamide mononucleotide. NR, nicotinamide riboside. Green arrows indicate enhanced or desired fluxes toward NMN biosynthesis, red crosses indicate blocked competing or degradation-associated reactions, and the purple module denotes a heterologous transport system introduced for pathway optimization.

However, systematic engineering of *P. putida* KT2440 for NMN biosynthesis has not been reported. In this study, we constructed an NMN-producing *P. putida* KT2440 strain through a “block–enhance–transport” strategy (Figure 1). First, the nicotinic acid (NA) degradation gene *nicB* and the putative NMN-consuming genes *pncC* and *ushA* were deleted. Second, the endogenous NAD^+^ de novo biosynthetic pathway was strengthened by overexpressing *nadA*, *nadB*, and *nadC*. Meanwhile, the heterologous Ft.nadE* from *Francisella tularensis* was also overexpressed. Third, a mutated *Salmonella enterica pnuC* transporter gene (Se.pnuC*) was integrated into the chromosome of *P. putida* KT2440 to facilitate NMN accumulation in the extracellular medium. The effects of further engineering of downstream NAD^+^ metabolism and PRPP supply were also evaluated. Overall, the engineered *P. putida* KT2440 strain produced extracellular NMN at a titer of 1.28 mM in shake-flask cultivation using glucose as a carbon source and L-aspartate as a precursor.

## 2. Materials and Methods

### 2.1. Strains, Plasmids, and Growth Conditions

The bacterial strains and plasmids used in this study are listed in Table 1 and Table 2, respectively. *E. coli* DH5α was used for plasmid construction and propagation. *P. putida* KT2440 was used as the parental strain for metabolic engineering. *E. coli* DH5α was cultivated in Luria–Bertani (LB) medium (10 g/L tryptone, 5 g/L yeast extract, and 10 g/L NaCl) at 37 °C, and *P. putida* strains were cultivated at 30 °C in LB medium for initial propagation. For shake-flask cultivation, basal salt medium (BSM) containing 14.06 g/L Na_2_HPO_4_·12H_2_O, 4 g/L KH_2_PO_4_, 1 g/L K_2_SO_4_, 0.2 g/L MgCl_2_, 4 g/L NH_4_Cl, and 1 mL/L trace element solution was used. The trace element solution contained 1 g/L CaCl_2_, 1 g/L FeCl_3_·6H_2_O, and 0.4 g/L MnCl_2_·4H_2_O. When indicated, glucose and potassium L-aspartate were added to the BSM at final concentrations of 10 g/L and 1 g/L, respectively. Where required, antibiotics were added at the following final concentrations: kanamycin (Kan), 50 μg/mL; chloramphenicol (Cm), 25 μg/mL; gentamycin (Gm), 50 μg/mL; and ampicillin (Amp), 100 μg/mL.

### 2.2. Construction of Chromosomal Gene Deletion Strains

The suicide vector pK18mobsacB was used for chromosomal gene deletion [26,27]. DNA fragments of approximately 600 bp located upstream and downstream of each target gene were amplified from the *P. putida* KT2440 genome and assembled into plasmid pK18mobsacB. The resulting deletion plasmids were introduced into *P. putida* KT2440 by conjugation using *E. coli* S17 as the donor strain. Single-crossover recombinants were selected on LB agar containing kanamycin and ampicillin. Double-crossover mutants were subsequently selected on LB agar supplemented with 15% (*w*/*v*) sucrose. Candidate colonies were verified by colony PCR using primers flanking the corresponding deletion regions. The genes *nicB*, *pncC*, and *ushA* were sequentially deleted to generate strain LW03. All primers used in this study are provided in Table S1. Genes involved in NMN biosynthetic pathways and their corresponding locus tags in *P. putida* KT2440 are listed in Table 3 [28].

### 2.3. Construction of Expression Plasmids and Chromosomal Integration of Se.pnuC*

The broad-host-range vectors pSEVA234 and pSEVA644 were used for gene expression in *P. putida* KT2440 [25]. The heterologous gene Ft.nadE* from *Francisella tularensis* [16], the transporter gene Se.pnuC* from *Salmonella enterica* [16], and endogenous genes including *pncB*, *nadA*, *nadB*, *nadC*, *nadD*, *nadE*, *mazG*, *zwfB*, *gntZ*, and *prs* were amplified and assembled into the corresponding vectors through homologous recombination (Table 3). To generate a chromosomally integrated Se.pnuC* expression module, an expression cassette containing the Ptrc promoter, lac operator, Se.pnuC*, and a transcriptional terminator was integrated into a selected intergenic site between PP_3732 and PP_3733 of strain LW03 using the pK18-::Se.pnuC* vector (Table 2). Correct integration was confirmed by colony PCR.

### 2.4. NMN Tolerance Assay

To evaluate NMN tolerance, wild-type *P. putida* KT2440 and strain LW04 were cultivated in BSM supplemented with 0–50 mM NMN and 10 g/L glucose as the sole carbon source. Cultures were incubated at 30 °C and 180 rpm for 60 h. Samples were collected every 12 h, and cell growth was monitored by measuring optical density at 600 nm (OD_600_).

### 2.5. Shake-Flask Cultivation

A single colony of each recombinant *P. putida* KT2440 strain was inoculated into LB medium containing the appropriate antibiotic and cultivated at 30 °C and 220 rpm for approximately 12 h. Cells were harvested, washed, and inoculated into 100 mL BSM with 10 g/L glucose in 500 mL shake flasks at an initial inoculum of 1% (*v*/*v*). The OD_600_ of each individual culture was monitored during cultivation. Because the growth rate varied among strains and independent cultivation batches, isopropyl-β-D-thiogalactopyranoside (IPTG) was added to each culture when its OD_600_ reached 0.3–0.4, rather than after a fixed cultivation period. At that point, IPTG and potassium L-aspartate were added to final concentrations of 1 mM and 1 g/L, respectively. Cultures were incubated at 30 °C and 180 rpm for up to 120 h. Samples were collected at 12 h intervals for cell growth and metabolite analysis.

### 2.6. HPLC Analysis of NMN and NA

For extracellular metabolite analysis, 1 mL cell cultures were centrifuged at 12,000 rpm for 2 min, and the supernatants were filtered through 0.22-μm aqueous membrane filters before HPLC analysis. For intracellular metabolite analysis, cell pellets from 1 mL cell cultures were washed twice with sterile water and resuspended in 1 mL of sterile water. The cell suspensions were disrupted on ice using a JY92-IIN sonicator (Scientz Inc., Ningbo, China) at 40% power in pulse mode (2 s on and 10 s off) until a cumulative sonication time of 1 min was reached. The resulting lysates were centrifuged at 12,000 rpm for 5 min, and the supernatants were filtered through 0.22-μm aqueous membrane filters before analysis.

NMN and NA were analyzed using an HPLC system equipped with a Shim-pack GIST C18-AQ column (250 mm × 4.6 mm, 5 μm, Shimadzu, Shanghai, China). The mobile phase consisted of 30 mM potassium phosphate buffer (pH 7.5) and methanol (95:5, *v*/*v*) and was delivered isocratically at a flow rate of 1.0 mL/min. The column temperature was maintained at 30 °C, the detection wavelength was set at 254 nm, and the injection volume was 10 μL. NMN and NA were identified by comparing their retention times with those of the corresponding authentic standards analyzed under identical HPLC conditions.

The identity of the peak assigned to NMN was further confirmed by liquid chromatography–mass spectrometry (LC–MS) using an Agilent 6546 LC/Q-TOF mass spectrometer (G6546A) equipped with a Polaris 5 C18-A column (150 mm × 4.6 mm, 5 μm, Agilent Technologies, Beijing, China). Mobile phases A and B were water containing 0.1% (*v*/*v*) formic acid and methanol, respectively. The flow rate was 0.5 mL/min. The gradient elution program was as follows: 0–1 min, 5% B; 1–12 min, 5–50% B; 12–15.5 min, 50–80% B; 15.5–25 min, 80% B; 25–30.5 min, 80–5% B; and 30.5–33 min, 5% B. Mass spectrometric detection was performed using an electrospray ionization (ESI) source operated in positive-ion mode over an *m*/*z* range of 200–440. NMN, which has a nominal molecular mass of 334 Da, was detected as the protonated molecular ion [M + H]^+^ at *m*/*z* 335.0641. The identity of NMN was confirmed by comparing its retention time and mass spectrum with those of an authentic NMN standard analyzed under the same conditions. The resulting LC–MS data are shown in Figure S1.

NMN and NA concentrations were quantified using external standard calibration curves (Figure S2). The linear ranges, regression equations, and coefficients of determination (*R*^2^) for both analytes are provided in Table S2. Representative chromatograms of authentic standard NMN and fermentation samples are presented in Figure S3. All cultivation experiments were performed with three biological replicates. Data are presented as mean ± standard deviation.

### 2.7. Statistical Analysis

Statistical analyses were performed using GraphPad Prism 9.5.1. Differences in NMN production among strains LW07–LW10 were analyzed using the Brown–Forsythe and Welch one-way ANOVA, followed by Dunnett’s multiple-comparison test, with LW07 used as the reference strain. Data are presented as mean ± SD from three independent biological replicates. A *p* value of less than 0.05 was considered statistically significant.

## 3. Results

### 3.1. Growth of Wild-Type P. putida KT2440 and Strain LW04 in NMN-Supplemented BSM

**Figure 2:**
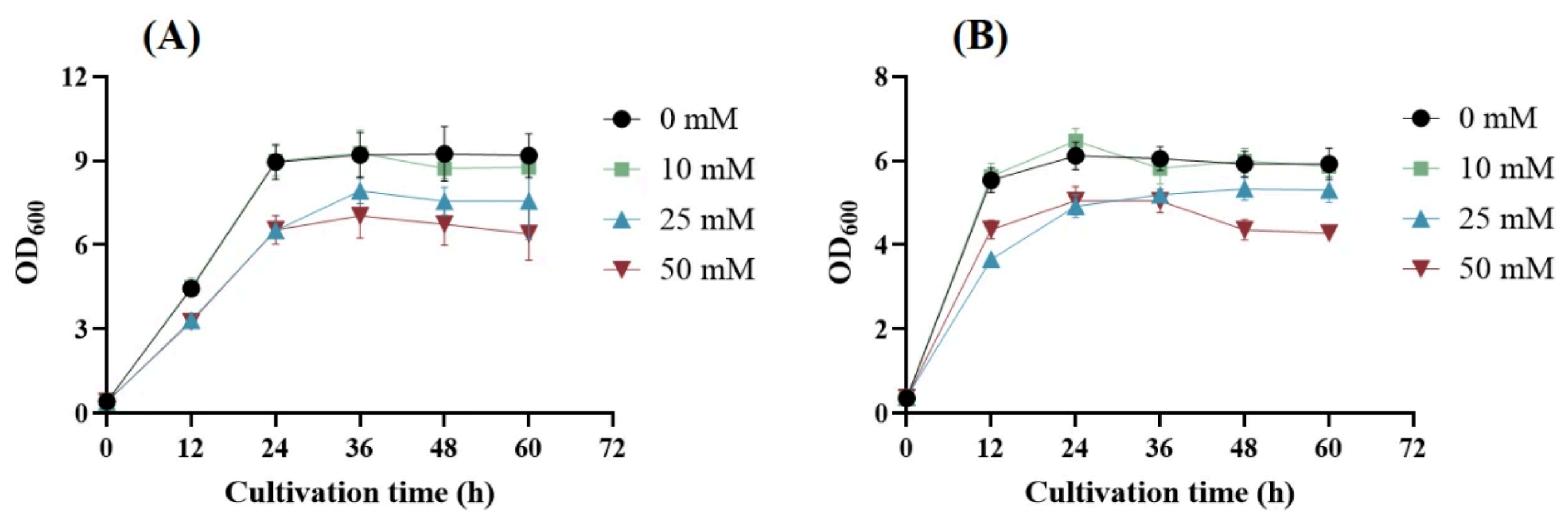
Growth curves of wild-type *P. putida* KT2440 and strain LW04. Strains were cultivated in BSM containing glucose (10 g/L) and different concentrations of NMN (0, 10, 25, and 50 mM): (**A**) wild-type *P. putida* KT2440; (**B**) strain LW04. Data are presented as mean ± SD from three biological replicates.

The growth of wild-type *P. putida* KT2440 and strain LW04 harboring the Se.pnuC* expression cassette was evaluated in BSM supplemented with 0–50 mM NMN. As shown in Figure 2, 10 mM NMN caused no apparent growth inhibition in either strain compared with the corresponding NMN-free control. At higher NMN concentrations, particularly 50 mM, both strains generally showed reduced biomass accumulation.

Although the two strains exhibited broadly similar responses to increasing NMN concentrations, LW04 generally reached lower cell densities than the wild-type strain after 24 h under the corresponding conditions, including in the absence of NMN. This difference suggests that LW04 has a reduced overall capacity for biomass accumulation under the tested culture conditions, potentially reflecting a basal fitness cost associated with strain construction or expression of the heterologous transporter. Because the lower cell density was also observed without NMN and the relative response to increasing NMN concentrations was similar in the two strains, the phenotype does not appear to result solely from increased NMN sensitivity. These results indicate that *P. putida* KT2440 possesses relatively high tolerance toward NMN under the tested conditions and may therefore be suitable for further development as an NMN-producing host.

### 3.2. Disruption of NA Degradation Enabled NA Accumulation

NA can serve as a precursor for NaMN biosynthesis through the action of nicotinate phosphoribosyltransferase, which is encoded by the *pncB* gene in *P. putida* KT2440 (Figure 1). NA can also be directed into a degradation pathway encoded by the *nic* gene cluster [28]. To reduce the metabolic flux toward NA degradation, the *nicB* gene was deleted from the genome of *P. putida* KT2440 using the plasmid pK18-Δ*nicB* (Figure S4), thereby generating strain LW01.

**Figure 3:**
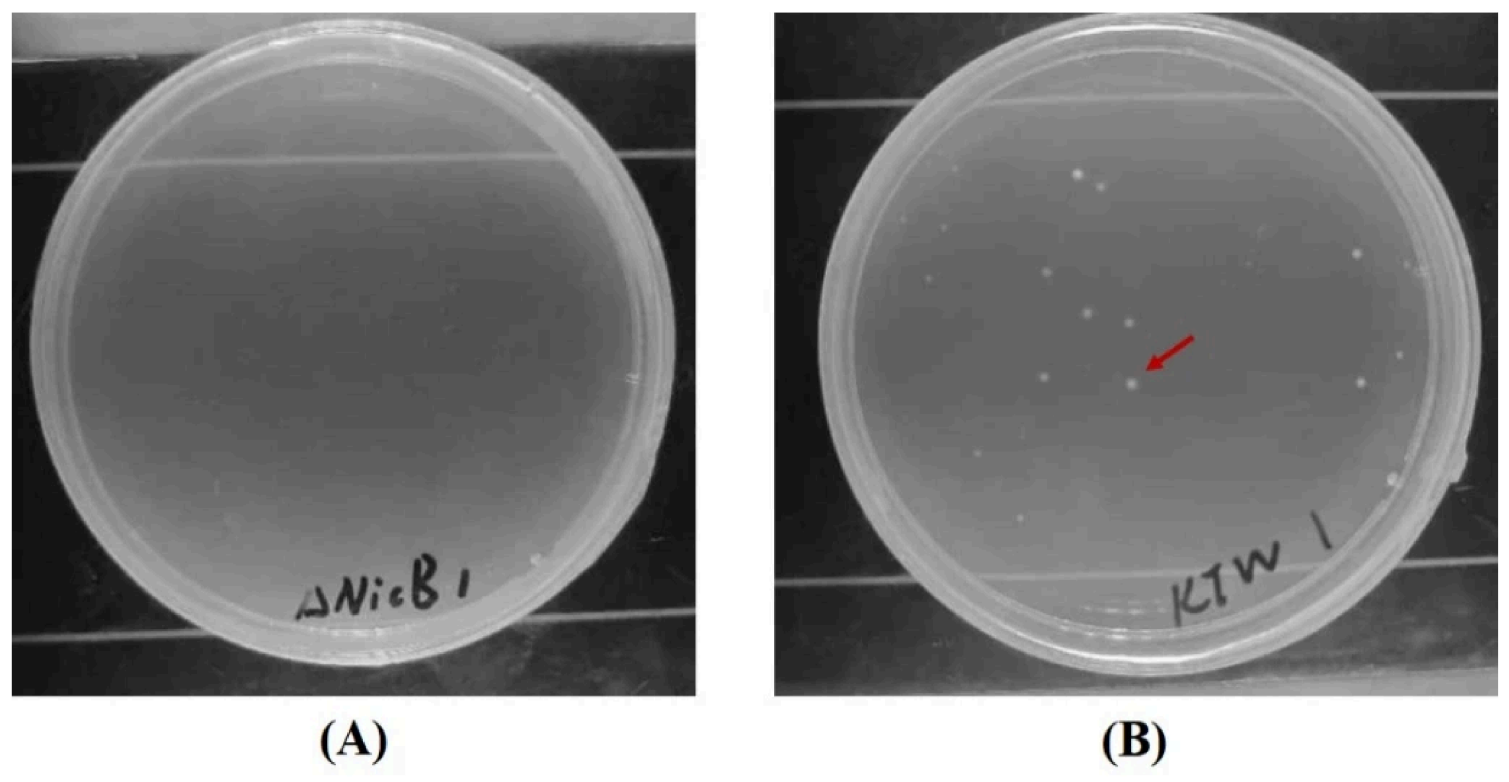
Effect of *nicB* deletion on nicotinic acid utilization. Strains were grown on BSM agar containing 10 g/L NA as the sole carbon source: (**A**) strain LW01; (**B**) wild-type *P. putida* KT2440. The red arrow indicates the growing cell colony.

Strain LW01 exhibited severely impaired growth on BSM agar containing NA (10 g/L) as the sole carbon source (Figure 3A), whereas the wild-type strain exhibited normal growth under the same conditions (Figure 3B). This growth phenotype confirmed that NA utilization was substantially disrupted in strain LW01.

Subsequently, strain LW01 was cultivated in shake flasks containing BSM supplemented with 10 g/L glucose and 1 g/L L-aspartate. After 84 h, the NA concentration in cell extracts reached 66.2 μM, whereas NMN remained undetectable. Although NMN was not detectable at this stage, these results indicated that deletion of *nicB* redirected a portion of the metabolic flux away from NA degradation and provided a foundation for subsequent pathway engineering.

### 3.3. Construction of a Chassis with Reduced Putative NMN Consumption

NMN can be metabolized through multiple reactions in *P. putida* KT2440. In this study, *pncC*, encoding a putative NMN amidohydrolase, and *ushA*, encoding a putative nucleotide phosphatase, were selected as candidate genes potentially involved in NMN consumption. Sequential deletion of *pncC* and *ushA* in the LW01 background generated strains LW02 and LW03, respectively (Figure S4).

Subsequently, strains LW02 and LW03 were cultivated in shake flasks containing BSM supplemented with 10 g/L glucose and 1 g/L L-aspartate. NMN accumulation was not detected in either strain. These results indicated that disruption of the selected NMN consumption pathways alone was insufficient to achieve detectable NMN accumulation, possibly because of insufficient endogenous metabolic flux toward NMN biosynthesis.

### 3.4. A Preiss–Handler Pathway-Based NA-to-NMN Route Enabled Low-Level NMN Formation

To establish an alternative route for NMN production, the heterologous Ft.nadE* gene from *Francisella tularensis* [16] was coexpressed with the endogenous *pncB* gene in strain LW03, generating strain LW05. This pathway was designed to promote the conversion of NA to NaMN by *pncB* and subsequently direct NaMN toward NMN formation via the heterologous enzyme.

After strain LW05 was cultivated for 120 h in BSM containing 10 g/L glucose and 10 g/L NA, a low-intensity HPLC peak assigned to NMN based on retention-time matching with an authentic NMN standard was observed. Quantification against the external NMN calibration curve corresponded to approximately 21 μM in the prepared cell extracts. The limited accumulation may have resulted from insufficient NaMN availability, suboptimal expression or catalytic activity of the enzyme Ft.nadE*, or competition with the native NAD^+^ biosynthetic pathway. Therefore, strengthening the endogenous NAD^+^ de novo biosynthetic pathway was considered a more promising approach for increasing the availability of the NaMN precursor.

### 3.5. Overexpression of nadABC Enabled NMN Accumulation

**Figure 4:**
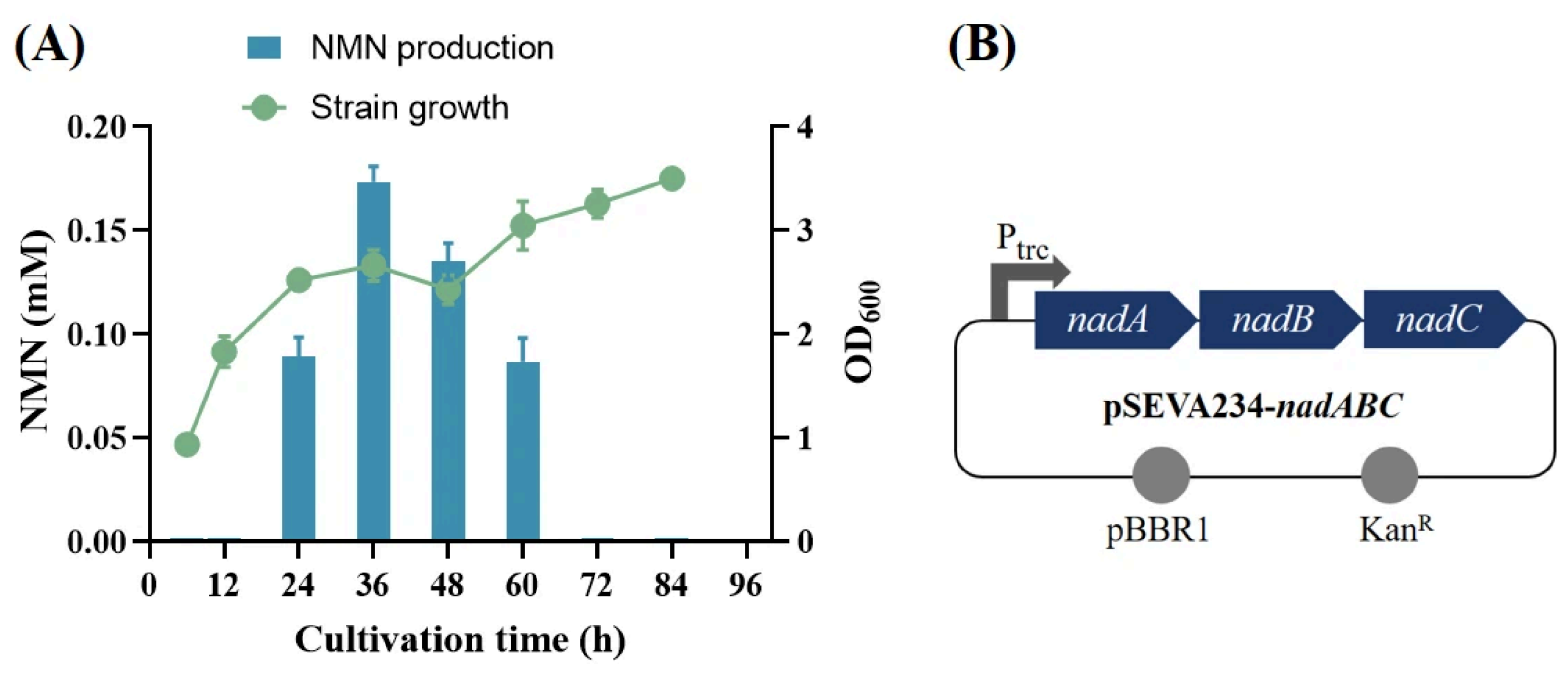
NMN accumulation and cell growth in strain LW06 overexpressing *nadABC*. NMN levels were determined in cell extracts prepared from fermentation samples: (**A**) Time-course profiles of intracellular NMN accumulation and cell growth during fermentation. (**B**) Schematic representation of the *nadABC* overexpression plasmid pSEVA234-*nadABC*.

The genes *nadA*, *nadB*, and *nadC* are involved in the conversion of L-aspartate into NaMN in the NAD^+^ de novo biosynthetic pathway in *P. putida* KT2440. To enhance precursor formation, these three endogenous genes were overexpressed in strain LW03. Based on HPLC-UV analysis and retention-time matching with the authentic standard, the resulting strain LW06 accumulated NMN to approximately 0.17 mM in the prepared cell extracts during shake-flask cultivation in BSM supplemented with 10 g/L glucose and 1 g/L L-aspartate (Figure 4). The extracellular accumulation of NMN in LW06 was not observed. Because the two pathway configurations in LW05 and LW06 were evaluated using different precursors and concentrations, their intrinsic efficiencies cannot be directly compared based on the present data. The decline in the NMN level after approximately 36 h may reflect further metabolism, degradation, changes in biomass-normalized accumulation, or other cellular processes.

### 3.6. Introduction of the Se.pnuC* Cassette Was Associated with Extracellular NMN Accumulation

A transporter module containing Se.pnuC* was previously introduced into *E. coli* to promote extracellular NMN accumulation [16]. Therefore, the Se.pnuC* expression cassette was integrated at a neutral chromosomal site in strain LW03, yielding strain LW04 (Figure S5). Subsequent expression of *nadABC* in this transporter-integrated background generated strain LW07, which exhibited substantially increased extracellular NMN accumulation.

**Figure 5:**
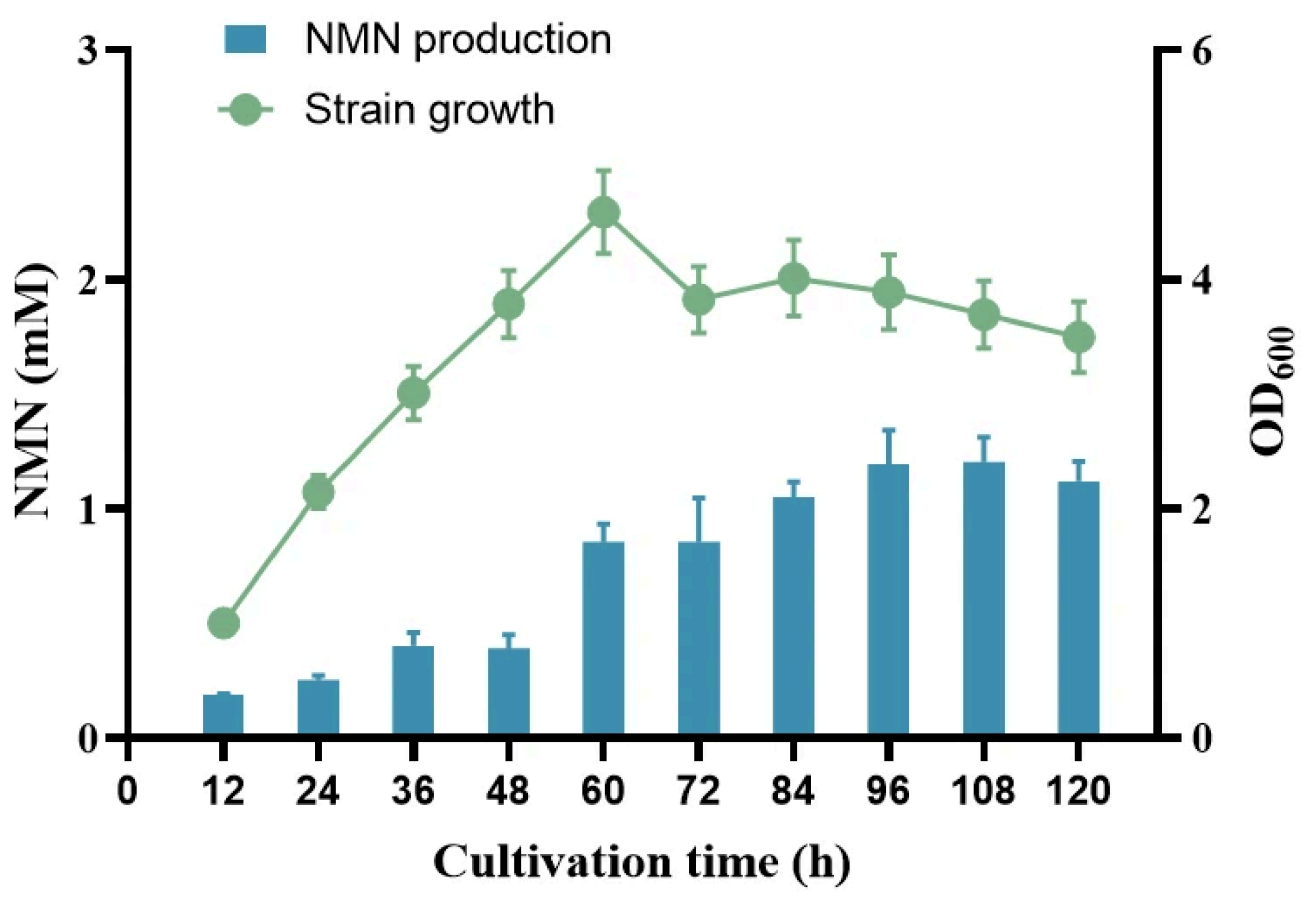
Extracellular NMN accumulation and cell growth profile of strain LW07 carrying chromosomally integrated Se.pnuC* and plasmid-borne *nadABC*.

As shown in Figure 5, LW07 achieved a maximum extracellular NMN concentration of 1.20 mM after 96 h of shake-flask cultivation in BSM supplemented with 10 g/L glucose and 1 g/L potassium L-aspartate. NMN was not detectable in the corresponding cell extracts under the analytical conditions used. Because NMN in LW06 and LW07 was measured in different sample fractions, the corresponding concentrations cannot be directly compared quantitatively. The extracellular accumulation observed in LW07 was associated with the introduction of the Se.pnuC* cassette; however, the current data do not distinguish between an effect on NMN transport, total biosynthetic flux, or other strain-dependent processes. A complete intracellular and extracellular NMN mass balance is required to establish the role of Se.pnuC*.

### 3.7. Additional Pathway Reinforcement Did Not Further Improve NMN Production

**Figure 6:**
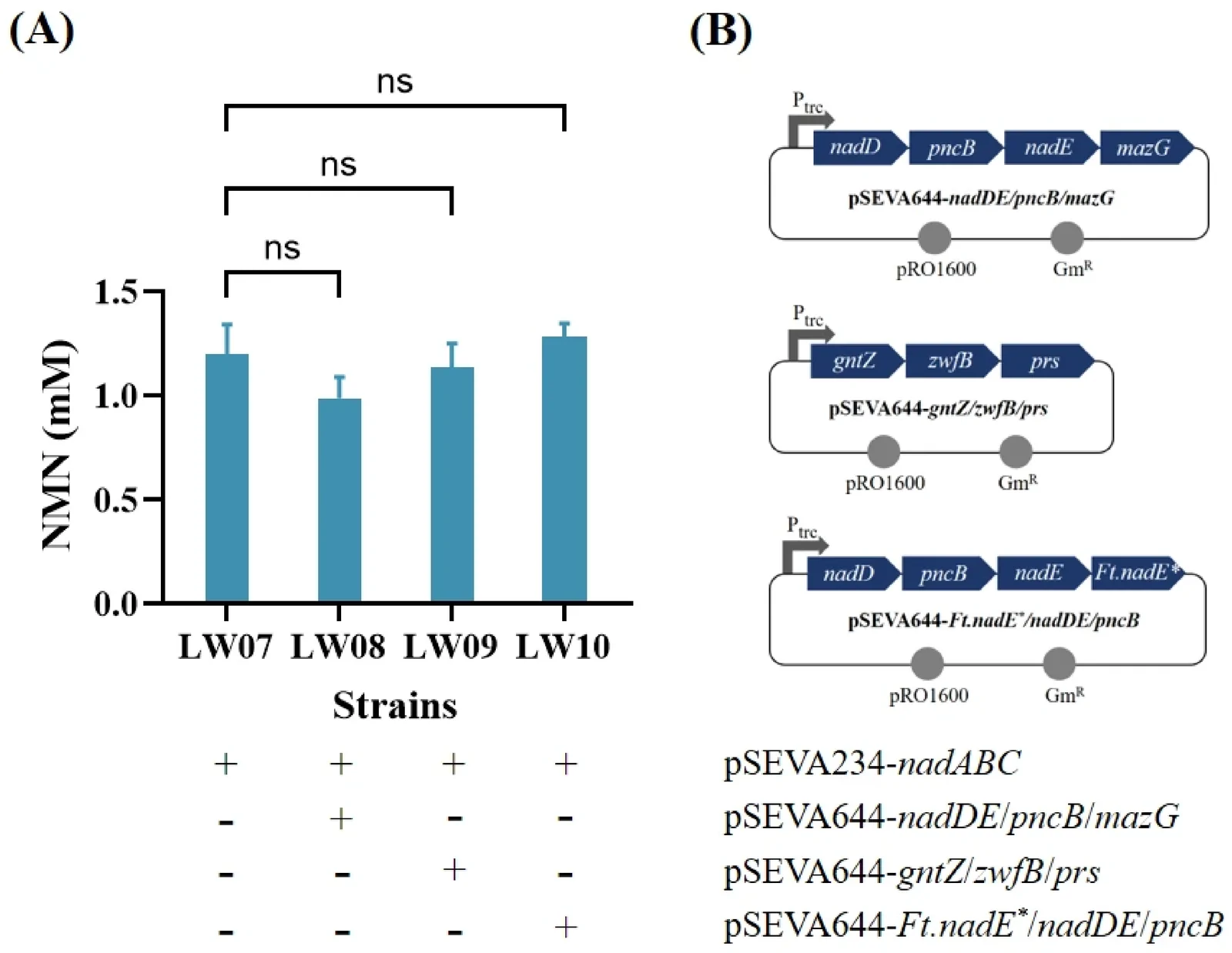
Comparison of extracellular NMN production among different strains and schematic representation of the corresponding plasmids: (**A**) Extracellular NMN accumulation in different strains. (**B**) Schematic representation of the plasmids carried by the corresponding strains. Statistical significance was assessed using the Brown–Forsythe and Welch one-way ANOVA followed by Dunnett’s multiple-comparison test, with LW07 as the reference strain. “ns” indicates no statistically significant difference compared with LW07 (*p* ≥ 0.05).

To further improve NMN production, genes involved in downstream NAD^+^ metabolism and PRPP supply pathways were overexpressed in the LW07 background. Overexpression of *nadD*, *nadE*, *pncB*, and *mazG* generated strain LW08. However, LW08 accumulated a maximum extracellular NMN concentration of 0.99 mM after 96 h, representing the lowest titer among the additionally engineered strains (Figure 6).

To enhance PRPP availability, the endogenous genes *gntZ*, *zwfB*, and *prs* were overexpressed, generating strain LW09. After 96 h, LW09 accumulated 1.13 mM extracellular NMN, which was close to the level achieved by LW07 (Figure 6).

A third strategy involved the overexpression of the heterologous gene Ft.nadE* together with *nadD*, *nadE*, and *pncB*, generating strain LW10. At 96 h, LW10 reached the numerically highest extracellular NMN concentration of 1.28 mM (approximately 0.43 g/L) (Figure 6).

These results suggest that the tested modifications of downstream NAD^+^ metabolism or PRPP supply did not substantially improve NMN production beyond the level achieved by LW07. Instead, excessive expression of these pathways may have imposed an additional metabolic burden, disrupted intracellular cofactor homeostasis, or redirected metabolic flux toward competing pathways.

## 4. Discussion

In this study, *P. putida* KT2440 was established as an alternative microbial chassis for the biosynthesis of NMN. The engineered strain LW10 produced 1.28 mM NMN (approximately 0.43 g/L) in the extracellular medium after 96 h of shake-flask cultivation. These results suggest the feasibility of redirecting NAD^+^ metabolism toward NMN production in *P. putida* KT2440. Given its metabolic versatility, stress tolerance, and well-developed synthetic biology toolbox [22,23,24], this organism may provide a useful complementary platform for NMN biomanufacturing.

Based on the final NMN concentration and the total cultivation time, the average volumetric productivity was calculated to be approximately 0.00448 g/L/h, equivalent to 4.48 mg/L/h. Residual glucose and L-aspartate concentrations were not measured during the shake-flask cultivation because of an oversight in the original experiments. Consequently, the substrate-based yield, calculated from the actual consumption of glucose and L-aspartate, could not be determined from the available data.

The systematic engineering results suggest that precursor supply through the NAD^+^ de novo pathway was a major constraint on NMN biosynthesis. Overexpression of *nadA*, *nadB*, and *nadC* resulted in an NMN concentration of approximately 0.17 mM in cell extracts, compared with approximately 21 μM obtained using the NA-fed Preiss–Handler pathway-based route involving endogenous *pncB* and engineered Ft.nadE*. Although these two routes were evaluated under different precursor-supplementation conditions, the results suggest that reinforcing quinolinate and NaMN formation from L-aspartate was more effective than the tested NA-dependent route in *P. putida* KT2440. This finding is consistent with previous studies showing that precursor availability and NaMN-to-NMN conversion efficiency are important determinants of microbial NMN production [29,30].

A second major improvement was associated with chromosomal integration of the engineered Se.pnuC* transporter cassette [16]. Strain LW06, which overexpressed *nadABC* in the deletion chassis, showed an NMN concentration of approximately 0.17 mM in cell extracts, whereas strain LW07, carrying both *nadABC* and the integrated Se.pnuC* cassette, accumulated 1.20 mM NMN in the extracellular medium after 96 h. NMN was not detectable in the cell extracts of LW07 under the analytical conditions used, a result compatible with facilitated extracellular NMN accumulation by the Se.pnuC* cassette [12,14,20,29,31,32,33]. Because NMN concentrations in the cell extracts and extracellular medium represent different sample fractions, these values cannot be directly compared quantitatively or used alone to establish an increase in total NMN biosynthetic flux. Nevertheless, the marked extracellular accumulation observed in LW07 indicates that the Se.pnuC* module played an important role in promoting NMN accumulation in the culture medium. It is also possible that transporter-mediated NMN translocation reduced intracellular product accumulation and thereby favored continued pathway activity; however, this hypothesis requires direct experimental validation. These results underscore the importance of transporters for extracellular NMN production, consistent with the previously reported application of Se.pnuC* in microbial production of NMN in various hosts [12,14,20,29,31,32,33].

Further reinforcement of downstream NAD^+^ metabolism or the PRPP supply pathway provided little or no additional improvement. Overexpression of *nadD*, *nadE*, *pncB*, and *mazG* in LW08 resulted in a maximum extracellular NMN titer of 0.99 mM, whereas overexpression of *gntZ*, *zwfB*, and *prs* in LW09 resulted in 1.13 mM NMN. LW10, expressing Ft.nadE* together with *nadD*, *nadE*, and *pncB*, achieved the highest observed titer of 1.28 mM, representing only a small numerical increase over the 1.20 mM obtained with LW07. These observations illustrate that increasing the expression of additional pathway enzymes does not necessarily result in a proportional increase in product formation. Several non-exclusive explanations may account for these observations, including expression burden, altered cofactor homeostasis, and diversion of NaMN toward native NAD^+^ metabolism [34]. In particular, increased *NadD* and *NadE* activities may favor the sequential conversion of NaMN to NaAD and NAD^+^ rather than NMN accumulation [18]. Likewise, overexpression of pentose phosphate pathway and PRPP biosynthesis genes may not increase effective PRPP availability if carbon flux, ATP supply, allosteric regulation, or competing nucleotide biosynthetic pathways remain limiting [21,35]. However, the present study did not quantify glucose or L-aspartate consumption, and NaMN, NaAD, NAD^+^, PRPP, and fermentation by-products were not measured. Therefore, the observed strain-dependent differences cannot be used to infer quantitative carbon-flux allocation, precursor conversion efficiency, PRPP limitation, or disruption of NAD^+^ homeostasis. The proposed effects of additional pathway-gene expression should be regarded as hypotheses requiring targeted metabolomic or isotope-tracing analysis.

Although the NMN titer achieved in this study remains lower than those reported for extensively optimized *E. coli* systems, which have reached up to 19.3 g/L under fed-batch conditions [12,36,37], direct quantitative comparisons should be made with caution because of differences in pathway design, precursor supplementation, cultivation mode, and process optimization. The current *P. putida* KT2440 platform is still at an early stage of development. In *E. coli*, integrated strategies involving heterologous nicotinamide phosphoribosyltransferase expression, PRPP supply enhancement, ATP regeneration, transporter engineering, and fed-batch optimization have enabled substantially higher NMN titers [11]. Beyond *E. coli*, NMN production has been demonstrated in engineered *Pichia pastoris*, *Lactococcus lactis*, and *Bacillus subtilis* through distinct biosynthetic routes [20,29,30]. Among these hosts, engineered *B. subtilis* achieved an NMN titer of up to 1.21 g/L during batch cultivation using NAM as the precursor [29]. NMN production has also been achieved in engineered *E. coli* and *Saccharomyces cerevisiae* through whole-cell biocatalysis using NR as the substrate [38,39], with a titer of up to 12.6 g/L reported for *S. cerevisiae* [38]. Collectively, these studies demonstrate the feasibility of NMN production in diverse microbial hosts. Accordingly, the primary contribution of the present study lies not in achieving a superior absolute titer but in establishing *P. putida* KT2440 as an alternative chassis for NMN biosynthesis.

Several limitations should be considered when interpreting the results presented in this study. Cultivation was performed primarily in shake flasks using a basal medium without systematic optimization, and strain performance under controlled bioreactor conditions remains unknown. In particular, glucose and L-aspartate supply should be optimized together with pH and dissolved oxygen because the strongly oxidative metabolism of *P. putida* KT2440 may make oxygen transfer a limiting factor at high cell density [40]. In addition, NMN concentrations in cell extracts were not normalized to cell dry weight, total protein content, or intracellular volume. Therefore, these values should be interpreted as concentrations in the prepared extracts rather than absolute intracellular concentrations. The direction and kinetics of Se.pnuC*-mediated NMN transport were also not directly determined. Future studies should quantify NMN mass balances across intracellular and extracellular fractions, assess cell integrity, and characterize transporter-dependent uptake and efflux.

Further improvement will require more precise balancing of NMN synthesis, native NAD^+^ metabolism, and product transport. Rather than broadly amplifying additional pathways, the expression of *nadABC*, Ft.nadE*, and Se.pnuC* should be optimized using promoter or ribosome-binding-site libraries, followed by stable chromosomal integration of the optimized modules. Partial downregulation of the competing NaMN branch, particularly the *nadD*-mediated conversion of NaMN to NaAD, may increase NaMN availability for NMN formation while preserving the NAD^+^ supply required for growth. A heterologous Nampt-dependent route could also be evaluated as a more direct pathway for converting nicotinamide and PRPP into NMN, but its performance should be compared with that of the current L-aspartate-derived route in terms of precursor requirements, competition for PRPP, and production cost. These targeted pathway-balancing and process-optimization efforts will help clarify the production potential of *P. putida* KT2440.

## 5. Conclusions

*P. putida* KT2440 was engineered to explore its potential as a host for NMN biosynthesis for the first time. Under the respective cultivation conditions tested, overexpression of *nadABC* in the deletion background resulted in the production of NMN in cell extracts, and extracellular NMN accumulation was observed in strains carrying the chromosomally integrated Se.pnuC* cassette. The highest extracellular concentration estimated by HPLC-UV was 1.28 mM after 96 h of shake-flask cultivation. However, the present data do not establish whether Se.pnuC* directly mediated NMN efflux or increased total NMN production. Meanwhile, additional reinforcement of downstream NAD^+^ metabolism or the PRPP supply pathway provided little or no further improvement, suggesting that balanced pathway expression is more important than indiscriminate gene overexpression. These findings establish *P. putida* KT2440 as an alternative chassis for NMN production and provide a basis for future pathway optimization, medium development, and bioreactor-scale validation.

## Figures and Tables

**Table 1:** Strains used in this study.

Strain	Description	Source
*E. coli* DH5α	Host strain for plasmid construction	Lab stock
*E. coli* S17	Conjugative donor strain for transfer of suicide vectors	Lab stock
*P. putida* KT2440	Wild-type strain, Amp^R^, Cm^R^	Lab stock
LW01	*P. putida* KT2440 Δ*nicB*	This study
LW02	*P. putida* KT2440 Δ*nicB* Δ*pncC*	This study
LW03	*P. putida* KT2440 Δ*nicB* Δ*pncC* Δ*ushA*	This study
LW04	LW03 carrying a chromosomally integrated Se.pnuC* expression cassette	This study
LW05	LW03 harboring pSEVA234-Ft.nadE*/*pncB*	This study
LW06	LW03 harboring pSEVA234-*nadABC*	This study
LW07	LW04 harboring pSEVA234-*nadABC*	This study
LW08	LW07 harboring pSEVA644-*nadDE*/*pncB*/*mazG*	This study
LW09	LW07 harboring pSEVA644-*gntZ*/*zwfB*/*prs*	This study
LW10	LW07 harboring pSEVA644-Ft.nadE*/*nadDE*/*pncB*	This study

**Table 2:** Plasmids used in this study.

Plasmid	Description	Source
pSEVA234	Vector for gene expression, Kan^R^	[25]
pSEVA644	Vector for gene expression, Gm^R^	[25]
pWB302	Vector carrying Se.pnuC* gene, Amp^R^	[16]
pLZ301	Vector carrying Ft.nadE* gene, Amp^R^	[16]
pK18mobsacB	Vector for selection of double crossover, Kan^R^, *sacB*	Lab stock
pK18-Δ*nicB*	pK18mobsacB derivative carrying the Δ*nicB* construct	This study
pK18-Δ*pncC*	pK18mobsacB derivative carrying the Δ*pncC* construct	This study
pK18-Δ*ushA*	pK18mobsacB derivative carrying the Δ*ushA* construct	This study
pK18-Δ*surE*	pK18mobsacB derivative carrying the Δ*surE* construct	This study
pK18-Δ*mazG*	pK18mobsacB derivative carrying the Δ*mazG* construct	This study
pK18-::Se.pnuC*	pK18mobsacB derivative carrying the Se.pnuC* chromosomal integration cassette	This study
pSEVA234-Ft.nadE*/*pncB*	pSEVA234 carrying expression cassettes for Ft.nadE* from *Francisella tularensis* and *pncB* from *P. putida* KT2440	This study
pSEVA234-*nadABC*	pSEVA234 carrying expression cassettes for *nadA*, *nadB*, and *nadC* from *P. putida* KT2440	This study
pSEVA644-*nadDE*/*pncB*/*mazG*	pSEVA644 carrying expression cassettes for *nadD*, *nadE*, *pncB*, and *mazG* from *P. putida* KT2440	This study
pSEVA644-*gntZ*/*zwfB*/*prs*	pSEVA644 carrying expression cassettes for *gntZ*, *zwfB*, and *prs* from *P. putida* KT2440	This study
pSEVA644-Ft.nadE*/*nadDE*/*pncB*	pSEVA644 carrying expression cassettes for Ft.nadE* from *Francisella tularensis* and *nadD*, *nadE*, and *pncB* from *P. putida* KT2440	This study

**Table 3:** Genes involved in NMN biosynthetic pathways and their corresponding locus tags in *P. putida* KT2440.

Gene Name	Predicted/Putative Function	Locus Tag in *P. putida* KT2440
*pncB*	Nicotinate phosphoribosyltransferase	PP_4868
*nadA*	Quinolinate synthase	PP_1231
*nadB*	L-aspartate oxidase	PP_1426
*nadC*	Quinolinate phosphoribosyltransferase	PP_0787
*nadD*	Nicotinate-nucleotide adenylyltransferase	PP_4810
*nadE*	NAD^+^ synthetase	PP_4869
*mazG*	Nucleoside triphosphate pyrophosphatase	PP_1657
*zwfB*	Glucose-6-phosphate 1-dehydrogenase	PP_4042
*gntZ*	6-phosphogluconate dehydrogenase	PP_4043
*prs*	Ribose-phosphate pyrophosphokinase	PP_0722
*nicB*	Nicotinate dehydrogenase subunit B	PP_3948
*pncC*	NMN amidohydrolase	PP_1628
*ushA*	5′-nucleotidase	PP_1414

## Data Availability

The original contributions presented in this study are included in the article/Supplementary Material. Further inquiries can be directed to the corresponding authors.

## Supplementary Materials

The following supporting information can be downloaded at https://www.mdpi.com/article/10.3390/microorganisms14091877/s1, Table S1: Primers used for plasmid construction and strain verification in this study; Table S2: Calibration parameters for NMN and NA; Figure S1: LC-MS analysis of NMN standard and fraction in fermentation sample: (A) TIC profile of NMN standard. (B) Mass spectra of NMN standard. (C) MIC profile of the peak assigned to NMN in fermentation sample. (D) Mass spectra of the peak assigned to NMN in fermentation sample; Figure S2: Calibration curves for the quantification of NMN and NA; Figure S3: HPLC–UV chromatograms of NMN standards and the sample. Chromatograms of NMN standards at concentrations of 0.020 mM (top) and 2.0 mM (middle) and the sample (bottom) containing three major chromatographic peaks. The NMN standards showed retention times of 3.397 and 3.411 min, respectively. The peak at 3.396 min in the sample was assigned to NMN based on its retention-time agreement with the NMN standards. Note that different absorbance and retention time scales are used in the three panels. AU, absorbance units; Figure S4: Verification of gene deletion mutants by colony PCR and agarose gel electrophoresis. (A) Verification of the Δ*nicB* mutant. M, 5000-bp DNA ladder; lanes 1–5, colony PCR products from candidate Δ*nicB* transformants. (B) Verification of the Δ*pncC* mutant. M, 5000-bp DNA ladder; lanes 1–4, colony PCR products from candidate Δ*pncC* transformants. (C) Verification of the Δ*ushA* mutant. M, 5000 bp DNA ladder; lanes 1–5, colony PCR products from candidate Δ*ushA* transformants. Red arrows indicate PCR products of the expected sizes, confirming successful gene deletion; Figure S5: PCR verification of the chromosomal integration of *Se.pnuC** by agarose gel electrophoresis. M, 5000 bp DNA ladder; lanes 1–8, PCR products from candidate positive transformants. Red arrows indicate PCR products of the expected size, confirming successful chromosomal integration of *Se.pnuC**.
