# Supplementary material for: Metabolic Engineering of Pseudomonas putida KT2440 for β-Nicotinamide Mononucleotide Biosynthesis from Glucose and Aspartate
Source: Microorganisms. 2026 Aug 24;14(9):1877. doi: 10.3390/microorganisms14091877 (PMC13609833; doi:10.3390/microorganisms14091877)
Supplement: Supplementary file 1 [file microorganisms-14-01877-s001.zip › microorganisms-4514640-supplementary.pdf]

Metabolic Engineering of *Pseudomonas putida* KT2440 for β–Nicotinamide Mononucleotide Biosynthesis from Glucose and Aspartate

Luna Gao [1], Lin Wei [1], Siqi Wang [1], Jun Li [2], Jingli Liu [1], Zhao Guo [1], Zhi-Min Li [3,*] and Zhimin Li [1,*]

[1] College of Bioscience and Bioengineering, Jiangxi Engineering Laboratory for the Development and Utilization of Agricultural Microbial Resources, Jiangxi Agricultural University, Nanchang 330045, China

[2] College of Food Science and Engineering, Jiangxi Agricultural University, Nanchang 330045, China

[3] College of Chemistry and Materials, Jiangxi Agricultural University, Nanchang 330045, China

* Correspondence: lzm_70@jxau.edu.cn (Z.-M.L.); zhiminli@jxau.edu.cn (Z.L.)

Tables

Table S1. Primers used for plasmid construction and strain verification in this study

Table S2. Calibration parameters for NMN and NA

Figures legend

Figure S1. LC-MS analysis of NMN standard and fraction in fermentation sample. (A) TIC profile of NMN standard; (B) Mass spectra of NMN standard; (C) MIC profile of the peak assigned to NMN in fermentation sample; (D) Mass spectra of the peak assigned to NMN in fermentation sample.

Figure S2. Calibration curves for the quantification of NMN and NA.

Figure S3. HPLC–UV chromatograms of NMN standards and the sample. Chromatograms of NMN standards at concentrations of 0.020 mM (top) and 2.0 mM (middle), and the sample (bottom) containing three major chromatographic peaks. The NMN standards showed retention times of 3.397 and 3.411 min, respectively. The peak at 3.396 min in the sample was assigned to NMN based on its retention-time agreement with the NMN standards. Note that different absorbance and retention time scales are used in the three panels. AU, absorbance units.

Figure S4. Verification of gene deletion mutants by colony PCR and agarose gel electrophoresis. (A) Verification of the Δ*nicB* mutant. M, 5,000-bp DNA ladder; lanes 1–5, colony PCR products from candidate Δ*nicB* transformants. (B) Verification of the Δ*pncC* mutant. M, 5,000-bp DNA ladder; lanes 1–4, colony PCR products from candidate Δ*pncC* transformants. (C) Verification of the Δ*ushA* mutant. M, 5,000-bp DNA ladder; lanes 1–5, colony PCR products from candidate Δ*ushA* transformants. Red arrows indicate PCR products of the expected sizes, confirming successful gene deletion.

Figure S5. PCR verification of the chromosomal integration of *Se.pnuC** by agarose gel electrophoresis. M, 5,000-bp DNA ladder; lanes 1–8, PCR products from candidate positive transformants. Red arrows indicate PCR products of the expected size, confirming successful chromosomal integration of *Se.pnuC**.

Table S1. Primers used for plasmid construction and strain verification in this study

**Primers**	**Sequences (5'-3')**
Δ*nicB*-UF	GAGCTCGGTACCCGGGGATCCCAAACAACCATCTCCCTGCAG
Δ*nicB*-UR	GTCAGGTCGACATGACCATTGAAG
Δ*nicB*-DF	AATGGTCATGTCGACCTGACCAAGCGTTCGAAGTGGTG
Δ*nicB*-DR	ACGACGGCCAGTGCCAAGCTTTTGAACGGGAAGCGCATC
Δ*pncC*-UF	GAGCTCGGTACCCGGGGATCCCGGATTGACGACTGGACGC
Δ*pncC*-UR	TCGATGTGGGTGCTCCTTGAGGGC
Δ*pncC*-DF	TCAAGGAGCACCCACATCGACGACAGGGGTTTGC
Δ*pncC*-DR	ACGACGGCCAGTGCCAAGCTTTGCATGTCACCCATCTCGC
Δ*ushA*-UF	GAGCTCGGTACCCGGGGATCCCAGGGCCGCTGATCTTCAA
Δ*ushA*-UR	AGAGGTAATGGCGATTGCGTACCCCACC
Δ*ushA*-DF	ACGCAATCGCCATTACCTCTTGAATGCCGGG
Δ*ushA*-DR	ACGACGGCCAGTGCCAAGCTTGGCCATTGCTGGTAGCGG
::*Se.PnuC**-UF	GAGCTCGGTACCCGGGGATCCACTTCCTCGACAACCCGCC
::*Se.PnuC**-UR	TGATGTCGGCGCATAAAGGTCGCGCCAGACC
::*Se.PnuC**-DF	TCCGGAACAACTGTCTTGCTCAAACG
::*Se.PnuC**-DR	ACGACGGCCAGTGCCAAGCTTGTGAGCAGGGCTACAGCGAA
::*Se.PnuC*-F*	ACCTTTATGCGCCGACATCATAACGGTTC
::*Se.PnuC*-R*	AGCAAGACAGTTGTTCCGGACCCCTGGATTCTCACCA
*Se.PnuC**-rbs	TGATAGCCTCCTTGCTCATGGATTTTTTTAGTACGCACAACATACT
*Se.PnuC**-F	GAGCTCGGTACCCGGGGATCCTGATAGCCTCCTTGCTCATGG
*Se.PnuC**-R	TCACGACGCGGCCGCAAGCTTTTAACGGGAAAGCGCGCG
*Ft.NadE**-rbs	TAAGAAGGAGATATACAATGAAAATCGTTAAGGATTTTAGCCCG
*Ft.NadE**-F	GAGCTCGGTACCCGGGGATCCTAAGAAGGAGATATACAATGAAAATCGTT
*Ft.NadE**-R	TCACGACGCGGCCGCAAGCTTTTAAAAGTTTGGGGTCAAAGCTAA
pncB-rbs	CGGCCGCGCGAATTCGAGCTCTTGATGAGCGAAAGCGCAT

Primers	Sequences (5'-3')
pncB-F	CGGCCGCGCGAATTCGAGCTCCGGCCGCGCGAATTCGAGC
pncB-R	TATATCTCCTTCTTATTACTCCTTGCTGGGGACTTTG
NadA-rbs	TAAGAAGGAGATATACAATGAGCCAACAATTCCAACATGATGT
NadA-F	GAGCTCGGTACCCGGGGATCCTAAGGAGGTTTTCTAATGACCCAGA
NadA-R	ATCAGGCATTACCAGACAGCTTCA
NadB-F	GCTGTCTGGTAATGCCTGATAAGAAGGAGATATACAATGAGCCAAC
NadB-R	TTCGGCATTAGAACCTCCTTAGCTCAGAGCGGGTTAAGGATGGTGTC
NadC-F	TTAACCCGCTCTGAGCTAAGGAGGTTCTAATGCCGA
NadC-R	TCACGACGCGGCCGCAAGCTTTCACAGGCTCAGTCGCATCG
NadD-F	CGGCCGCGCGAATTCGAGCTCGCTAAGGAGGTTCTAATGGCCA
NadD-R	CTCATCAATAGAAAACCTCCTTATCAGTTGGGCGCACGATATAGT
pncB-F	TAAGGAGGTTTTCTATTGATGAGCGAAAGCGCATTC
NadE-R	TATATCTCCTTCTTAGGATCCTCAAGGAGCGAACGGCAGT
mazG-F	CCGTTCGCTCCTTGAGGATCCTAAGAAGGAGATATACAATGACCTACACCCTTG ACGAC
mazG-R	TCACGACGCGGCCGCAAGCTTTCAGCCGCAGCTGGGCAG
pSEVA-F	GCGCGTTGGCCGATTCATTA
pSEVA-R	GGGACCCCTGGATTCTCACC
pK18-F	CATTAGGCACCCCAGGCTTT
pK18-R	CAGGGTTTTCCCAGTCACGA

Table S2. Calibration parameters for NMN and NA

**Parameter**	**NMN**	**NA**
Linear range (mM)	0.01–2.00	0.05–10.0
Regression equation	y = 3009186x -78621	y = 2271003x-56100
Coefficient of determination (R^2)	0.9916	0.9960

Note: x represents the analyte concentration (mM), and y represents the chromatographic peak area (mAu•s).

(A)

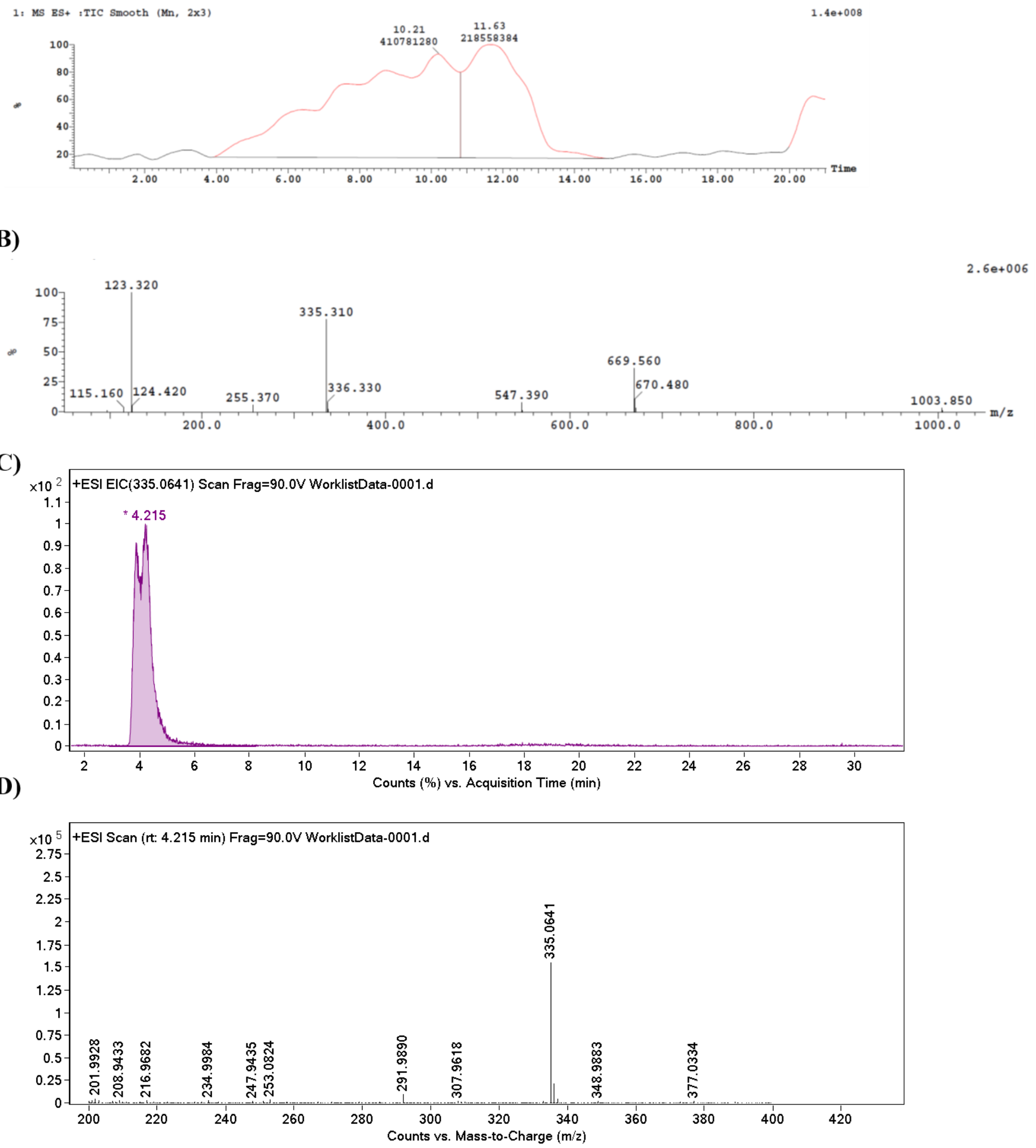

Figure S1. LC-MS analysis of NMN standard and fraction in fermentation sample. (A) TIC profile of NMN standard; (B) Mass spectra of NMN standard; (C) MIC profile of the peak assigned to NMN in fermentation sample; (D) Mass spectra of the peak assigned to NMN in fermentation sample.

(A) (B)

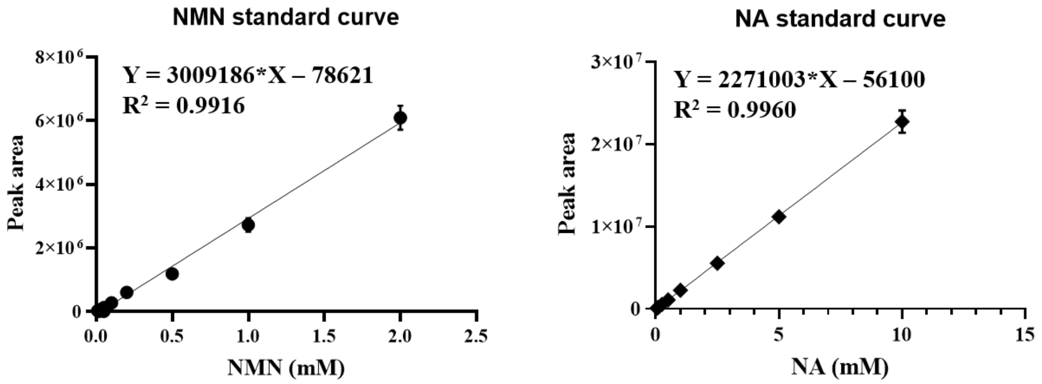

Figure S2. Calibration curves for the quantification of NMN and NA.

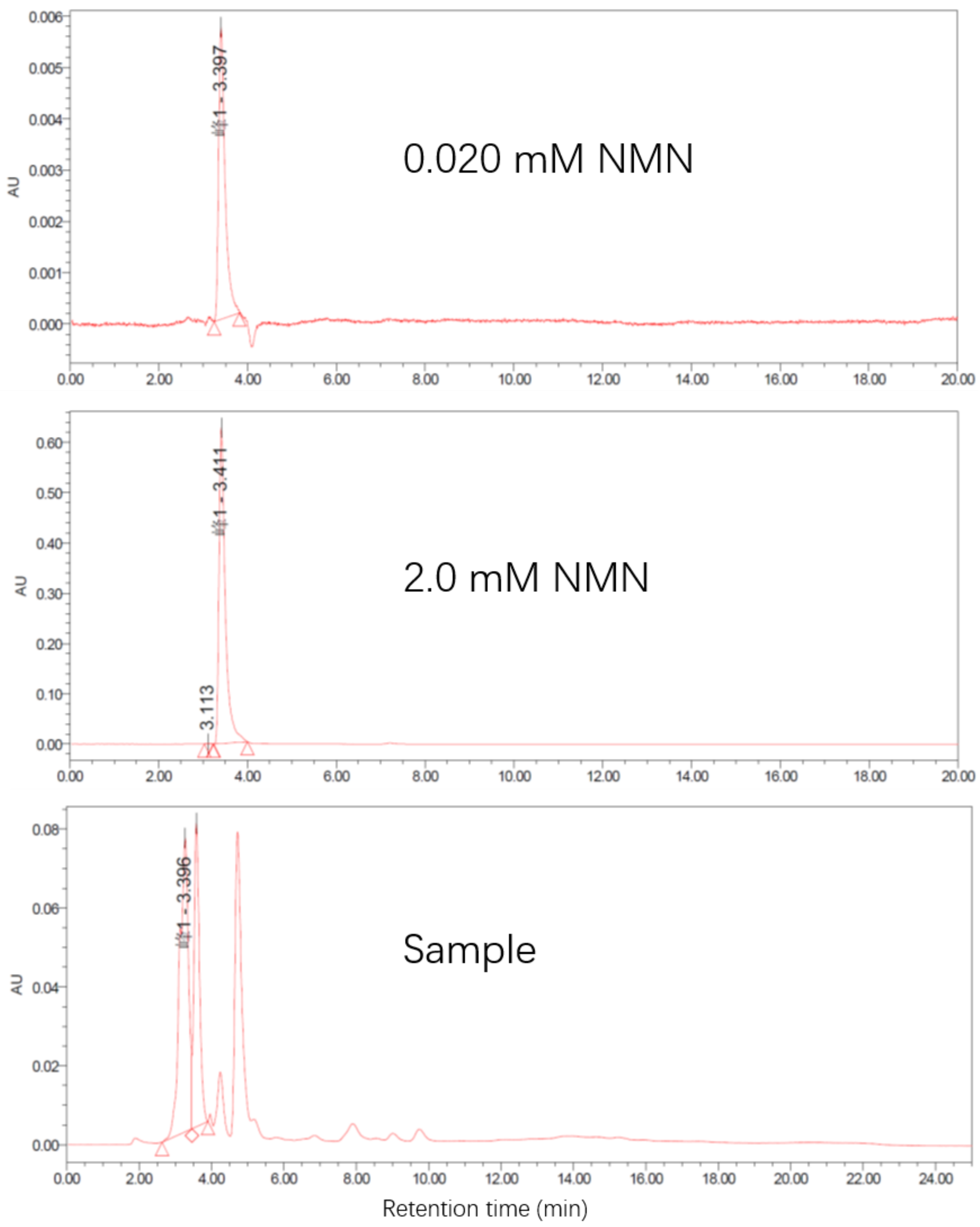

Figure S3. HPLC–UV chromatograms of NMN standards and the sample. Chromatograms of NMN standards at concentrations of 0.020 mM (top) and 2.0 mM (middle), and the sample (bottom) containing three major chromatographic peaks. The NMN standards showed retention times of 3.397 and 3.411 min, respectively. The peak at 3.396 min in the sample was assigned to NMN based on its retention-time agreement with the NMN standards. Note that different absorbance and retention time scales are used in the three panels. AU, absorbance units.

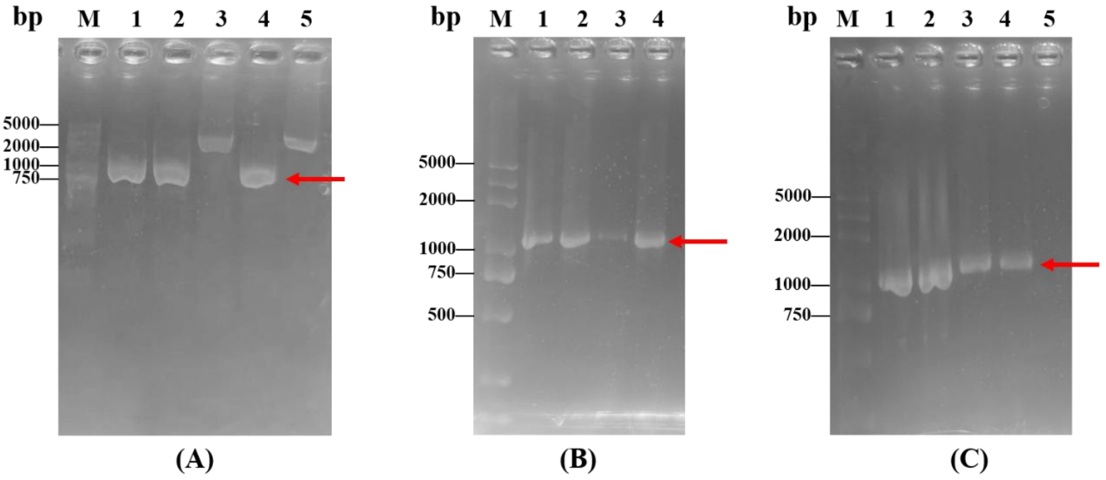

Figure S4. Verification of gene deletion mutants by colony PCR and agarose gel electrophoresis. (A) Verification of the Δ*nicB* mutant. M, 5,000-bp DNA ladder; lanes 1–5, colony PCR products from candidate Δ*nicB* transformants. (B) Verification of the Δ*pncC* mutant. M, 5,000-bp DNA ladder; lanes 1–4, colony PCR products from candidate Δ*pncC* transformants. (C) Verification of the Δ*ushA* mutant. M, 5,000-bp DNA ladder; lanes 1–5, colony PCR products from candidate Δ*ushA* transformants. Red arrows indicate PCR products of the expected sizes, confirming successful gene deletion.

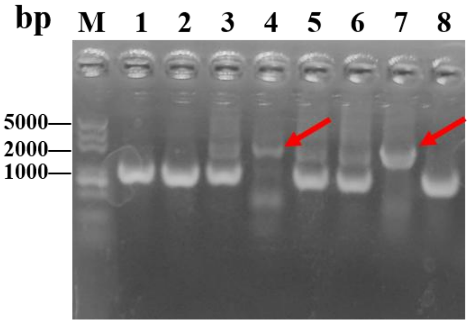

Figure S5. PCR verification of the chromosomal integration of *Se.pnuC** by agarose gel electrophoresis. M, 5,000-bp DNA ladder; lanes 1–8, PCR products from candidate positive transformants. Red arrows indicate PCR products of the expected size, confirming successful chromosomal integration of *Se.pnuC**.
